# Supplementary material for: Macrophages Treated with VEGF and PDGF Exert Paracrine Effects on Olfactory Ensheathing Cell Function
Source: Cells. 2022 Aug 4;11(15):2408. doi: 10.3390/cells11152408 (PMC9368560; doi:10.3390/cells11152408)
Supplement: Supplementary file 1 [file cells-11-02408-s001.zip › cells-1818336-supplementary.pdf]

SUPPLEMENTARY FILES

Table S1. Forward and Reverse primer sequences used in qPCR assay.

Gene Name	Forward sequence	Reverse sequence	Reference
Tnfa	GCCTCTTCTCATTCCTGCTTG	CTGATGAGAGGGAGGCCATT	[33]
Il6	CCAGTTGCCTTCTTGGGACT	GGTCTGTTGGGAGTGGTATCC	
Jun	GAACTGCATAGCCAGAACAC	GTTGAAGTTGCTGAGGTTGG	
Sox2	GCCATTAACGGCACACTGC	CCCCTCCCAATTCCCTTGTA	
Ngfr	AGCCCTCAAGGGTGATGGC	CCTCGTGGGTAAAGGAGTCTATATG	
Bdnf	TGCAGGGGCATAGACAAAAGG	CTTATGAATCGCCAGCCAATTCTC	
Gdnf	TGACCAGTGACTCCAATATGCC	CCGCTTGTTTATCTGGTGACCT	
Egr2	AGGCCCCTTTGACCAGATGA	AAGATGCCCGCACTCACAAT	
Pou3f1	CGCCAAGCAGTTCAAGCAA	TTGAGCAGCGGTTTGAGCTT	
Sox10	TCAAGAAGGAACAGCAGGAC	CTTTCGTTCAGCAACCTCCAG	
Mpz	CCCTGGCCATTGTGGTTTAC	CCATTCACTGGACCAGAAGGAG	
Srebf1	CCTGCTTGGCTCTTCTCTTT	CTGGTGCAGCTTATGGTAGAC	
Actin	CGTGCGTGACATCAAAGAGAA	TGGATGCCACAGGATTCCAT	

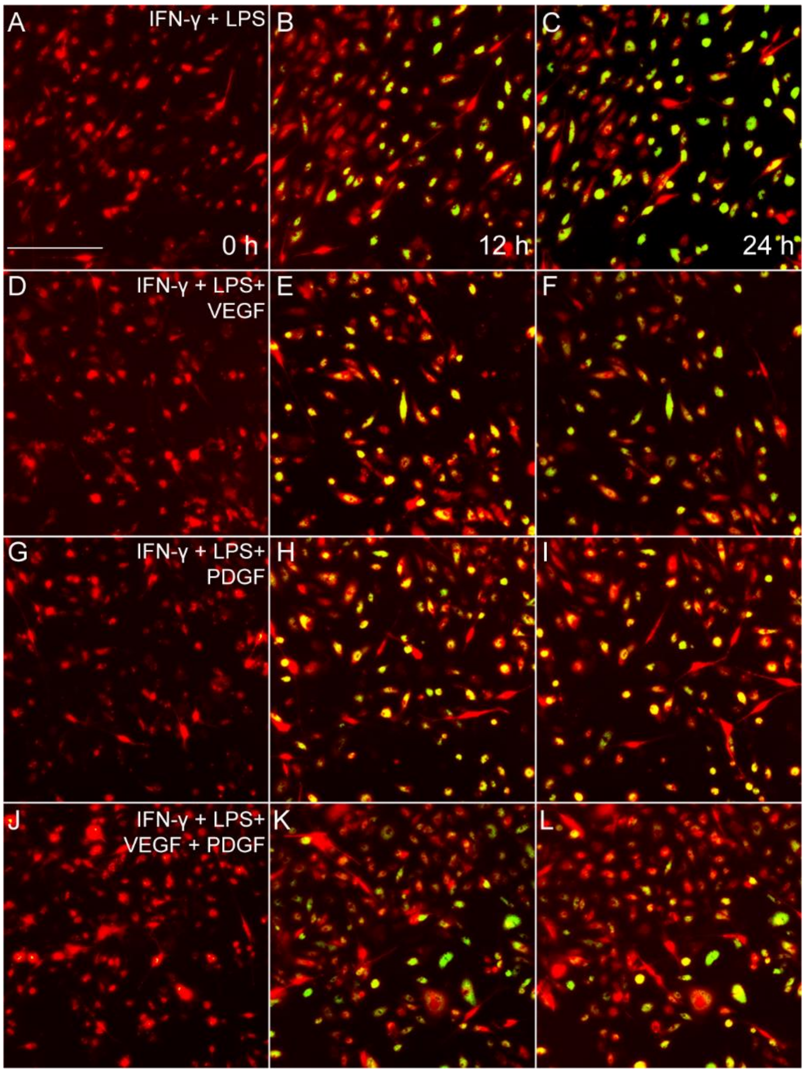

Figure S1. Representative time lapse images (Incucyte) of OECs (DsRed) phagocytosing myelin debris (green objects) when exposed to 24 h MCM containing inflammatory media with growth factors at 0, 12 and 24 h. **(A-C)** IFN-γ + LPS at 0 h **(A)**, 12 h **(B)**, 24 h **(C)**; IFN-γ + LPS + VEGF at 0 h **(D)**, 12 h **(E)**, 24 h **(F)**; IFN-γ + LPS + PDGF at 0 h **(G)**, 12 h **(H)**, 24 h **(I)**; IFN-γ + LPS + VEGF + PDGF at 0 h **(J)**, 12 h **(K)**, 24 h **(L).** Scale bar represents 200 μm for all images in the panel.
